# Direct Healthcare Costs Associated with Oligoarticular Juvenile Idiopathic Arthritis at a Single Center

**DOI:** 10.1155/2020/5640425

**Published:** 2020-09-01

**Authors:** Amit Thakral, Daniel Pinto, Michael Miller, Megan L. Curran, Marisa Klein-Gitelman, Dustin D. French

**Affiliations:** ^1^Department of Pediatrics, Emory University School of Medicine, Atlanta, GA, USA; ^2^Department of Physical Therapy, College of Health Sciences, Marquette University, Milwaukee, WI, USA; ^3^Department of Medical Social Sciences, Feinberg School of Medicine, Northwestern University, Chicago, IL, USA; ^4^Department of Pediatrics, Feinberg School of Medicine, Northwestern University, Chicago, IL, USA; ^5^Department of Pediatrics, University of Colorado School of Medicine, Aurora, CO, USA; ^6^Department of Ophthalmology, Feinberg School of Medicine, Center for Healthcare Studies, Feinberg School of Medicine, Northwestern University, Chicago, IL, USA; ^7^Veterans Affairs Health Services Research and Development Service, Chicago, IL, USA

## Abstract

Oligoarticular juvenile idiopathic arthritis (JIA) is a common disease in pediatric rheumatology. The management of oligoarticular JIA can result in a considerable economic burden. This study is a four-year, retrospective cost identification analysis performed to determine the annual direct cost of care for patients with oligoarticular JIA and possible predictive clinical factors. Direct healthcare costs were defined as those associated with office visits, laboratory studies, hospital admissions, joint injections, medications, infusions, radiology tests, and emergency room visits. Disease characteristics and patient information included ANA status, gender, age at diagnosis, duration from diagnosis to initial visit during the study period, and whether uveitis had been diagnosed. We identified 97 patients with oligoarticular JIA eligible for the study. The median age of diagnosis was 4.3 years. Positive ANA were noted in 75% of patients. 34% of patients received at least one intra-articular steroid injection. 32% of patients were prescribed a biologic during the study period, predominantly with other medications, while 23% of patients received only NSAIDs. 20% of patients were prescribed oral steroids. The average total direct medical cost in this study per year for an oligoarticular JIA patient was $3929 ± 6985. Medications accounted for 85% of annual direct medical costs. Clinic visits and laboratory testing accounted for 8% and 5%, respectively. Patient characteristics and demographics were tested for association with direct medical costs by the Wilcoxon rank sum test and Kruskal-Wallis test. Patients who were ANA positive had increased annual costs compared to patients who are ANA negative. ANA-positive patients were found to have statistically significant costs, particularly, in laboratory tests, procedural costs, radiology costs, and medication costs. The results reported here provide information when allocating healthcare resources and a better understanding of the economic impact oligoarticular JIA has on the United States healthcare system.

## 1. Introduction

Juvenile idiopathic arthritis (JIA) is characterized by inflammation and damage to the joints and affects approximately 1 in 1,000 children worldwide [1]. Being the most common rheumatic disease in childhood, JIA is a chronic disease that can have a serious impact on quality of life even when controlled [2]. Little is known about the economic impact of oligoarticular JIA [3]. According to the International League of Associations for Rheumatology (ILAR), JIA is classified into seven categories based on clinical and laboratory features that present in the first 6 months [4]. Oligoarticular JIA, one of the most common JIA subtypes, affects four or fewer joints during the first 6 months of illness [5]. Oligoarticular JIA is further divided into two categories, persistent and extended, depending on the number of joints involved after the initial six-month period. Patients are considered to have persistent oligoarticular JIA if they continue to have four or fewer joints after the first 6 months. Extended oligoarticular JIA patients have more than 4 joints affected after the first 6 months of symptoms.

Evolving health care reform requires managing direct costs of care incurred by different treatment modalities for children with arthritis and other chronic illnesses. The multidisciplinary management of oligoarticular JIA, including frequent hospital visits, laboratory evaluation, and medical treatments, can result in a considerable economic burden. Data on the cost of JIA are less available than for other illnesses [6]. With the expanded use of biologic agents, drug costs are beginning to replace inpatient care as the greatest source of direct medical costs [7]. The economic impact of JIA and whether it is associated with particular clinical characteristics are not well known [3]. The aim of this study is to determine the annual direct cost of care for oligoarticular JIA patients and identify any predictors of increased costs.

## 2. Methods

A four-year, retrospective cost identification analysis was performed to determine the annual direct cost of care for patients with oligoarticular JIA and possible predictive clinical factors.

### 2.1. Patients

Patients meeting ILAR criteria for the diagnoses of persistent and extended oligoarticular juvenile idiopathic arthritis [4] between January 2011 and December 2014 followed by the Division of Pediatric Rheumatology at Ann & Robert H. Lurie Children's Hospital of Chicago were identified by extracting diagnosis codes from the electronic medical record (EMR). Those seen less than yearly were excluded, as they were assessed as being in remission, no longer needing follow-up visits, or lost to follow-up. Patients who carried an ICD code diagnosis of oligoarticular JIA were reviewed to ensure they met criteria for diagnosis. Disease characteristics and patient information extracted from each patient's record included ANA status, gender, age at diagnosis, duration from diagnosis to initial visit during the study period, age at time of first visit, and whether uveitis had been diagnosed. Counts of active arthritic joints at the start and end of the study period were obtained. We also extracted data identifying which patients had joint injections and whether these injections were done in the clinic, operating room, or interventional radiology department.

### 2.2. Cost Data

Health care costs were measured as a direct cost to the hospital for acquisition of materials and services. Direct healthcare costs were defined as those associated with office visits, laboratory studies, hospital admissions, procedures such as joint injections, medications, day hospital infusions, radiology tests, and emergency room visits. All costs were obtained by the hospital's business analytics department.

Costs of outpatient medications were calculated using a combination of prescriptions sent, (extracted from the EMR) and formulary pricing. Costs from infusions received at Lurie Children's Hospital were obtained via the business analytics department; infusion center visits were considered hospital encounters. Our study did not include infusions done at other centers or through home health since those costs could not be obtained.  Table 1 summarizes the patient population for this study. Data are expressed as number (percentage) or median (interquartile range).

### 2.3. Statistical Analyses

All statistical analyses were performed using SAS statistical software (Release 9.4, SAS Institute, Cary, NC). Percentages, median (with interquartile range), or mean ± SD were used where appropriate to express disease characteristics, patient demographics, and direct medical costs. The Wilcoxon rank sum test was used to determine correlations between patient characteristics and direct medical costs.

## 3. Results

### 3.1. Patient Characteristics

We identified 97 patients (92% females) with oligoarticular JIA eligible for the study. The median age of diagnosis and median duration from diagnosis (in 2011) for patients in our study were 4.3 and 8.2 years, respectively. Positive anti-nuclear antibodies were noted in 75% of patients. 34% (33 patients) had received at least one intra-articular steroid injection. Of the patients who received intra-articular joint injections, 62% (21 patients) had joint injections performed by interventional radiology, whereas 30% (10 patients) had joint injections in the operating room, and 6% (2 patients) in our outpatient clinic, respectively. 27% (26 patients) had a diagnosis of uveitis documented in our EMR. 76% of patients were classified as having persistent oligoarticular JIA. A medication profile of the 97 patients is shown in Table 2. 32% of patients were prescribed a biologic during the four-year study period, predominantly in conjunction with other medications, while 23% of patients received only NSAIDs over the four-year period. 20% of patients were prescribed oral steroids during the study period.

### 3.2. Annual Direct Medical Costs and Its Components

Table 3 summarizes annual direct medical costs for patients in our study by cost components. The average total direct medical cost in this study per year for an oligoarticular JIA patient was $3929 ± 6985. Medications (including infusions) accounted for 85% of annual direct medical costs. Clinic visits and laboratory testing accounted for 8% and 5%, respectively. On average, annual clinic visits were $332 with a standard deviation of $312.

### 3.3. Predictors of Direct Medical Costs

Patient characteristics and demographics were tested for association with direct medical costs by the Wilcoxon rank sum test for all two group comparisons and the Kruskal-Wallis test for all comparisons with three or more groups. Variables that were evaluated for associations with direct costs included ANA, gender, diagnosis of uveitis, subtype of oligoarticular JIA, and intra-articular steroid injections.  Table 4 lists the annual direct costs ± SD as well as *p* values for the associated variable. The ANA status and oligoarticular JIA subtype were statistically significant, with *p* values of 0.02 and 0.04, respectively. Patients who were ANA positive had increased annual costs compared to patients who are ANA negative. ANA-positive patients were found to have statistically significant costs, particularly, in laboratory tests (*p* = 0.08), procedural costs (*p* = 0.04), radiology costs (*p* = 0.06), and medication costs (*p* = 0.02). Extended oligoarticular JIA patients had higher costs than patients classified as persistent oligoarticular JIA subtype. Extended oligoarticular JIA patients particularly had statistically significant medication costs (*p* = 0.04) compared to persistent oligoarticular JIA patients, with medication costs of $3932 ± 3943 and $2613 ± 4725, respectively. When evaluating costs for the number of arthritic joints at the start and end of the study, an increase or decrease in joint count over four years detected no difference in annual total direct cost.

## 4. Discussion

JIA is a complex chronic condition associated with a considerable economic burden to families affected as well as the health care system. We investigated costs associated with oligoarticular JIA. In our study, the mean annual total cost of oligoarticular JIA was estimated as $3929 ± 6985. As expected, the cost of medications, including infusions, (85%) was the largest contributor for costs. Extended oligoarticular JIA had a statistically significant higher mean annual cost of care than persistent oligoarticular JIA patients. ANA positivity was also found to be associated with higher annual costs of care. This result could be explained by the association of ANA positivity with uveitis, resulting in more aggressive treatment plans, often including biologic agents.

Our study had cost component results similar to other cost studies of JIA patients. In previously published studies, the majority included and categorized costs by the various subtypes of JIA. Our findings are similar to studies finding that patients with JIA have high costs, particularly, those related to office visits, medication costs, and diagnostic testing [3]. An assessment by Minden et al. found that the mean total direct cost of care in patients with active JIA of any subtype was approximately 3000 euros per year ($3282 in 2017 US dollars) [1]. Current data shows that biologics are reducing direct and indirect healthcare costs if one excludes the costs of the drug itself since the cost of biologics are ten times as high as conventional drug treatment [8]. A study done in Turkey found that medications contributed to 90% of health care costs associated with JIA, where anti-TNF drugs were the major source of these costs [9]. A United Kingdom study provided JIA costs during the first year of diagnosis and found that the total direct costs were 3205 US dollars and 3881 US dollars for oligoarticular persistent and oligoarticular extended JIA patients, respectively [10]. This study also found that costs significantly vary according to JIA subtype. Ours is the first study to explore differences in JIA subtype and ANA status that were associated with different costs.

Results of costing have been similar in studies for other rheumatologic conditions. In a South Korean cost study for systemic lupus erythematous patients, the largest component of direct medical costs were medications, with a mean annual cost amounted to 3305 US dollars in 2010 [11]. In adult rheumatoid arthritis (RA), a recent study found that costs attributable to RA-specific therapies, particularly, biologic therapies, represented almost half of direct four-year costs [7].

Our study was subject to several limitations. First, data was primarily extracted from the EMR and was dependent on proper diagnosis coding, physical examination documentation, procedure coding, and updated medication lists. Provider variability in medication treatment plans and laboratory evaluations could lead to variability in costs. Micro costing was performed on prescription drugs ordered in the EMR, which were assumed to be filled. Costs extracted were limited to services provided within our institution. Outside costs such as laboratory studies and infusions were not included, although most oligoarticular JIA patients do not receive infusions. This study only evaluated direct costs, not indirect costs such as time missed from school, transportation costs, or work absences for guardians, which could also pose a significant economic burden. In a United Kingdom study, it was found that JIA is likely to have a considerable economic impact, especially with regards to nonhealth care costs due to its societal impact [6]. We also did not have complete data on uveitis severity, because patients were often followed by local ophthalmologists, with notes variably sent to us. This study characterized patients with oligoarthritis. Future studies will explore the relation of economic burden to disease severity and evolving practices that includes telemedicine visits, and evolving new therapies.

## 5. Conclusion

In summary, we identified the annual direct medical costs for oligoarticular JIA. The results reported here provide information when allocating healthcare resources and a better understanding of the economic impact oligoarticular JIA has on the U.S. healthcare system. Even though oligoarticular JIA is considered one the more mild subtypes of JIA, it is still associated with high costs. Our study found that direct medical costs were higher in oligoarticular JIA patients who were ANA positive or had an extended course and that medications account for over 80% of direct medical costs in patients with oligoarticular JIA. The costs involved with the care of JIA patients are important for physicians and policy makers to consider when making informed decisions about patient care, especially as targeted drug therapies become more widely available for children.

## Figures and Tables

**Table 1:** Patient characteristics (*n* = 97).

Total number of patients	97
Female (%)	92
Median age of diagnosis (yrs)	4.3 (2.5-7.9)
Median duration from diagnosis (yrs) in 2011	8.2 (5.6-11.6)
ANA positivity (%)	75
Patients that received intra articular joint injections (%)	34
Patients with uveitis (%)	26
Persistent oligoarticular JIA (%)	76
Extended oligoarticular JIA (%)	24

**Table 2:** Medication profile over a 4-year period (*n* = 97).

	Number of patients	% of patients
NSAIDs only	21	23
NSAIDs and methotrexate	19	20
NSAIDs, biologics, and methotrexate	13	13
NSAIDs, sulfasalazine, and methotrexate	9	9
NSAIDs, biologic, methotrexate, and oral steroids	8	8
NSAIDs and sulfasalazine	7	7
Other (combination of the above)	20	20
Total	97	100

**Table 3:** Annual direct medical costs for patients with oligoarticular JIA (*N* = 97).

Costs	Average cost per person, standard deviation (% total direct annual cost)
Clinic visits	332 ± 312 (8)
Laboratory testing	197 ± 217 (5)
Radiology	34 ± 123 (1)
Procedural	45 ± 122 (1)
Medications	2927 ± 4567 (75)
Infusions	394 ± 2212 (10)
Total direct costs	3929 ± 6985 (100)

Data are expressed as number (percentage) or mean ± standard deviation (SD).

**Table 4:** Annual direct mean costs based on patient characteristics (via Wilcox rank sum test).

	Cost per patient	*p* value
ANA		0.02
ANA positive	4415 ± 7709	
ANA negative	2418 ± 3050	
Gender		0.49
Female	4027 ± 7136	
Male	2734 ± 3182	
Intra-articular steroids		0.82
Received	2642 ± 1777	
Not received	4580 ± 8340	
Oligoarticular JIA subtype		0.04
Persistent	3671 ± 7527	
Extended	4722 ± 4311	
Uveitis		0.19
Diagnosis of uveitis	3574 ± 7718	
No diagnosis of uveitis	4041 ± 3716	
Infusions		0.0007
Infusions received	25786 ± 19057	
No infusions	2732 ± 2550	

## Data Availability

The cost data used to support the findings of this study are available from the corresponding author upon request.
